# A 23‐million‐year record of morphological evolution within Neotropical grass pollen

**DOI:** 10.1111/nph.20214

**Published:** 2024-10-27

**Authors:** Caixia Wei, Mao Li, Limi Mao, Luke Mander, Phillip E. Jardine, William D. Gosling, Carina Hoorn

**Affiliations:** ^1^ Institute for Biodiversity and Ecosystem Dynamics University of Amsterdam Amsterdam 1090GE the Netherlands; ^2^ Donald Danforth Plant Science Center Saint Louis 63132 MO USA; ^3^ Key Laboratory of Palaeobiology and Petroleum Stratigraphy, Nanjing Institute of Geology and Palaeontology Chinese Academy of Sciences Nanjing 210008 China; ^4^ School of Environment, Earth and Ecosystem Sciences The Open University Milton Keynes MK7 6AA UK; ^5^ Institute of Geology and Palaeontology University of Münster Münster 48149 Germany

**Keywords:** evolution, morphospace, Neogene, pollen diversity, quantitative image analysis

## Abstract

- Grass‐dominated biomes in South America comprise *c*. 20 million years of history, yet their evolution and underlying drivers remain poorly understood.
- Here we apply a novel approach that combines scanning electron microscopy imaging with computational analysis to quantify the morphometrics of grass (Poaceae) pollen micro‐ornamentation from the Neotropics since the Early Miocene (23 million years ago). Three spatial–temporal pollen sets were assembled to further elucidate the variation and evolutionary traits of grasses through space and time.
- Our results reveals that three spatial–temporal pollen groups occupy unique, partially overlapping regions of their exine morphospace. The direction of this shift is consistent over time, progressing towards less dense ornamentation. Interestingly, the extent of the occupied morphospace did not vary significantly. This is the first time that the true morphological variation in Poaceae pollen micro‐ornamentation becomes apparent through time.
- We hypothesize that changes in grass pollen exine since the Early Miocene were driven by evolutionary processes (evolutionary drift and/or directional selection), and potentially migration at the continental scale. The high diversity in pollen micro‐ornamentation is likely related to their evolutionary success in the Neogene.

## Abstract 2

See also the Commentary on this article by Punyasena, **246**: 8–11.

## Introduction

The grasses (Poaceae) are one of the most diverse angiosperm families on Earth, comprising close to 12 000 species (Dahlgren *et al*., 1984; Soreng *et al*., 2022). Today grassy biomes cover over a quarter of the planet's land surface, including temperate grasslands, tropical savannas, and croplands (Gibson, 2009; Blair *et al*., 2014), with open grasslands playing a crucial role on the evolution of grazing mammals through the Cenozoic (Janis *et al*., 1998; Strömberg, 2011). Moreover, the evolution and development of modern humans (genus *Homo*) was profoundly intertwined with the emergence of the grassy savannas for over 2 million years (Strömberg & Staver, 2022), and ultimately, this led to agricultural societies emerging through the domestication of grasses (Linder *et al*., 2018). Today, grasses are an important crop plant and supply humans with building material and biofuels (Strömberg, 2011). The ecological and economic importance of grasses means that this plant group has received considerable attention in an effort to understand the mechanisms behind its evolutionary history and geographical expansion (Jacobs *et al*., 1999; Strömberg, 2011; Jaramillo, 2023).

Phytolith and molecular phylogenetic studies form the backbone of our knowledge about the evolutionary history of grasses. The fossil record of phytoliths (silica bodies produced by plants that are taxonomically diagnostic) suggests that the origin of grasses dates backed to the Cretaceous (*c*. 100 million years ago) (Prasad *et al*., 2005; Poinar *et al*., 2015; Wu *et al*., 2018). Molecular phylogenetic studies suggests that multiple independent diversification events occurred within the grasses leading to the development of C_4_ photosynthesis from C_3_ ancestors since the Paleocene (Huang *et al*., 2022). However, our understanding of grass evolution is limited because grass phytoliths are not always present in the fossil record, and the molecular clock estimates are limited by a reliance on extant taxa and therefore cannot evaluate the many species that went extinct over time.

Despite the high abundance of grass pollen in the sedimentary record, fossil grass pollen grains have not yet been used to reconstruct the diversification history and paleoecology of grasses (Mander & Punyasena, 2015; Hoorn *et al*., 2017; Jaramillo *et al*., 2017; Kirschner & Hoorn, 2020). This is because any morphological differences between the pollen grains of different grass taxa are difficult to observe using transmitted light microscopy, which is the traditional tool of researchers counting large numbers of fossil pollen grains for the purpose of reconstructing vegetation (Page, 1978; Beug, 2004; Bush, 2022). Being able to reconstruct changes in grass assemblage composition from the fossil record would allow us to compare the grasses with other major plant groups in the same sediment samples, and provide a potential counterpoint to phytolith and molecular records of the history of grassy biomes. The fossil record of grass pollen therefore represents a potentially rich source of information on the history of grasses that is currently underexplored.

### Approach and possible scenarios in Poaceae (pollen) evolution

**Fig. 1:**
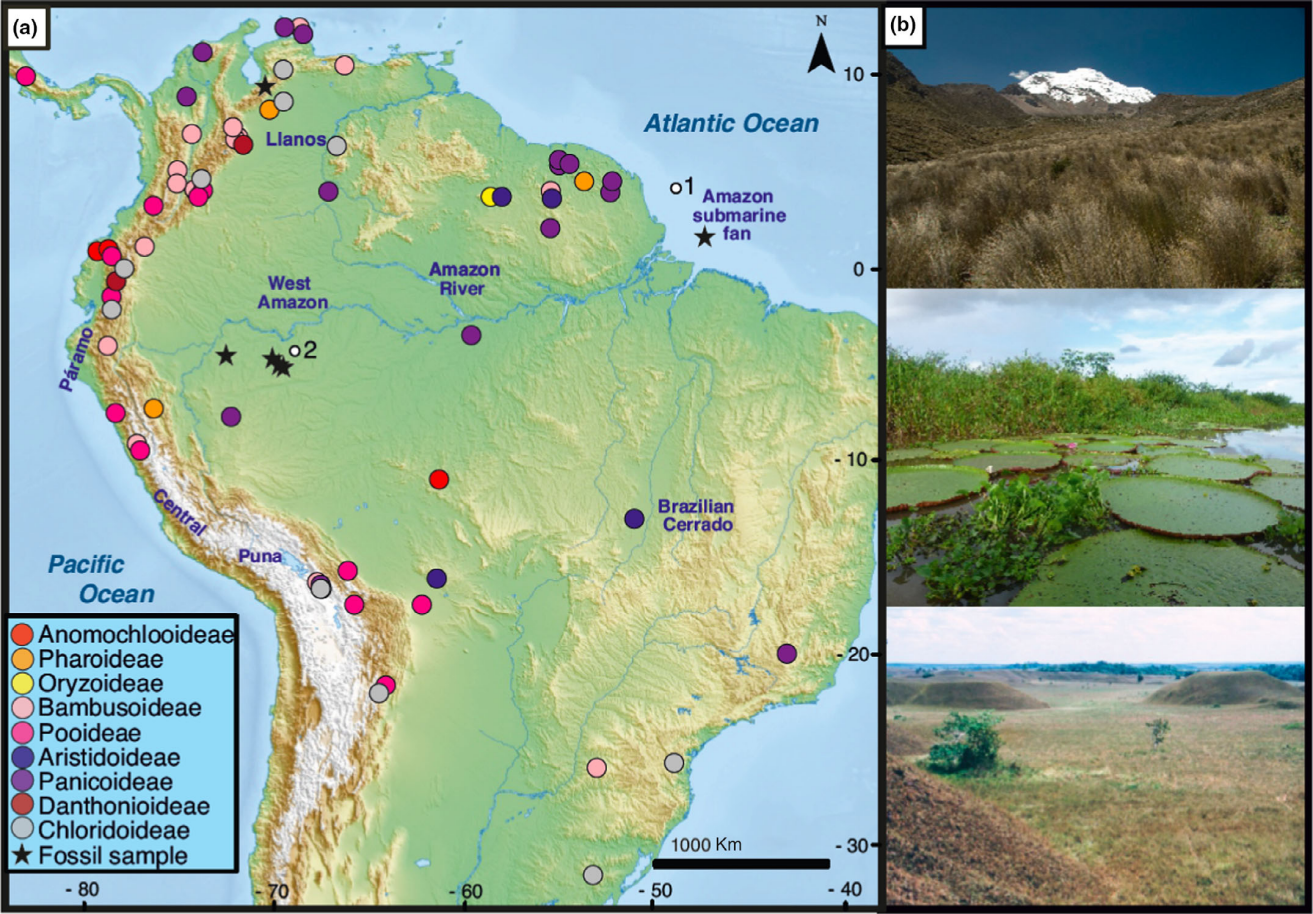
(a) Study sites of Poaceae fossil and extant pollen grains. Solid circles represent the collection localities for the extant herbarium specimens and inner circle colors denote the different grass subfamilies; black pentagrams showed the locations of fossil samples. The base map was downloaded from https://mapswire.com. (b) Different grasses habitats in present South America, from upper panel to lower panel: paramo grassland near the Chimborazo volcano in Ecuador (image credits: Esteban Suarez); grasses of the várzea near Manaus, Brazil (image credits: Carina Hoorn); savannas terrestrial of Llanos Orientales, Colombia (image credits: Henry Hooghiemstra).

In this paper we investigate the problem of the unresolved Poaceae pollen morphology by using a combination of scanning electron microscopy (SEM) and computational image analysis to investigate the micro‐morphological change in grass pollen since *c*. 23 Ma in South America. Our study includes the Early Miocene to Pleistocene fossil pollen from the western Amazon, the Amazon submarine fan (Brazil) and the Maracaibo Basin (Venezuela), and extant pollen from across the grass phylogeny and a variety of ecosystems across South America (Fig. 1). We use SEM to reveal morphological details on the surfaces of grass pollen grains (Mander & Punyasena, 2015; Page, 1978; Andersen & Bertelsen, 1972; Köhler & Lange, 1979), and use computational image analyses rooted in graph theory to quantify the morphological variation among these grass pollen grains (Mander *et al*., 2013).

**Fig. 2:**
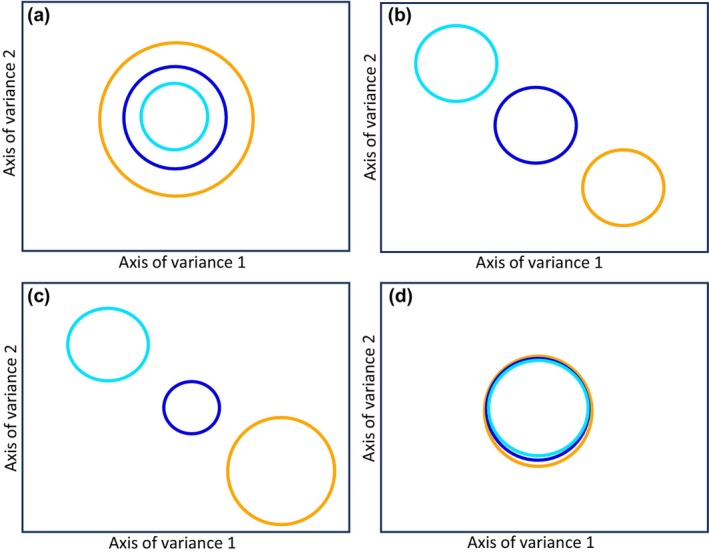
Four possible scenarios for changes in grass pollen morphospace occupation through time in South America. Each colored circle represents the extent and location of morphospace occupancy in multi‐dimensional space for a distinct time period (cyan circle = Early to Middle Miocene; dark blue circle = Late Miocene to Pleistocene; orange circle = extant). Scenario 1: expansion of grass pollen morphospace occupation through time (a). Scenario 2: change only in grass pollen morphospace location (but not extent) through time (b). Scenario 3: change both in grass pollen morphospace extent and location through time (c). Scenario 4: stability of morphospace through time (d).

Our microscale observations of pollen morphology are aimed at characterizing differences between grass pollen grains by quantifying the pollen morphological space (morphospace). We envision four potential scenarios for changes in grass pollen morphospace occupancy over time in South America and the likely mechanism driving change in our study region:

In Scenario 1, we envision a shift in the extent of morphospace occupation over time. Here, we anticipate a gradual expansion of the morphospace as time passes. This pattern could be explained by the evolution, or immigration, of taxa with different pollen morphology. If this hypothesis is supported, it is expected that the morphospace of extant pollen will encompass the entire range of the fossil morphospace (Fig. 2a). In Scenario 2, we foresee a shift in the location of morphospace occupation over time, but little change in the extent of the morphospace occupied. In this model we propose that there has either been an evolutionary drift leading to a gradual change in morphology, or balanced directional selection (extinction vs evolution/immigration) (Fig. 2b). In Scenario 3, we anticipate a shift in both the extent and location of the morphospace occupation. In this model we propose that directional selection, extinction, or immigration contributes strongly to changes in pollen morphology. For instance, a pulse of evolution, or a wave of new taxa arriving, into the area would result in a large expansion and change in position of the morphospace occupancy, that is due to geological events and/or climate change (Fig. 2c). Finally, in Scenario 4, we anticipate no expansion in either the extent, or location, of the morphospace occupation. The morphospace occupancy of grass pollen remains highly conserved over time (Fig. 2d).

## Materials and Methods

### Collection of specimens

#### Extant specimens

The Poaceae subfamily contains 12 subfamilies at the global level (Soreng *et al*., 2022). We restricted our research to 68 plant specimens from nine subfamilies across the phylogeny and ecosystems in northern South America. Pollen was extracted from these specimens collected from the National Herbarium of the Netherlands (Naturalis) (L) (Supporting Information Dataset S1). All extant sample information is available through previous research (Wei *et al*., 2023).

#### Fossil materials

A total of 19 grass fossil samples were obtained from the Early Miocene to the Pleistocene in South America, including nine samples from fluviolacustrine deposits in the western Amazon, nine marine samples from the Amazon submarine fan and one from Venezuela (the Maracaibo Basin) sample. The sample age and locality information are documented in previous studies (Hoorn, 1994; Boonstra *et al*., 2015; Bermúdez *et al*., 2017; Hoorn *et al*., 2017, 2019, 2022) (Dataset S2).

### Pollen processing

#### Extant pollen

The extant pollen grains were collected from anthers of herbarium specimens and processed with standard acetolysis and stored in glycerin jelly (Erdtman, 1952; Faegri *et al*., 1989). The pollen grains were transferred from glycerin to ethanol for SEM imaging with a gradual concentration series (70%, 96% and 100%). The pollen processing methods are documented in Wei *et al*. (2022, 2023).

#### Fossil pollen

The fossil pollen was retrieved from Neogene samples of the Amazon drainage basin. The western Amazon materials and the samples from Venezuela were treated by Na_4_P_2_O_7_, heavy liquid separation (clastics) and Schultze reagent (lignite) (Hoorn, 1994; Boonstra *et al*., 2015; Bermúdez *et al*., 2017; Hoorn *et al*., 2019, 2022). The Amazon submarine fan materials were processed with HCl, Na_4_P_2_O_7_, acetolysis and heavy liquid separation (Hoorn *et al*., 2017; Jardine *et al*., 2021). All fossil pollen were stored in glycerin after processing. The fossil pollen grains were transferred from glycerin to ethanol with a gradual concentration series (70%, 96% and 100%), or single grains were directly from glycerin (Zetter, 1989; Halbritter *et al*., 2018) ahead of scanning electron microscopy (SEM) imaging.

### Scanning electron microscopy (SEM) imaging

Dehydrated fossil and extant pollen grains were transferred to SEM stubs and coated with Platinum. We imaged pollen using SEM (TESCAN MAIA3, Czech (NIGPAS, China); Zeiss Gemini Sigma 300 FEG SEM (AMC, Amsterdam, the Netherlands)). Nine to 24 grains were examined and imaged for each extant specimen. One to 42 grains were imaged for each fossil sample depending on the pollen richness. Each pollen grain was imaged under three magnification levels: ×15 000, ×75 000, and ×150 000. All images were saved in high resolution for morphological analysis. The extant pollen images were made available through previous research (Wei *et al*., 2023).

### The fossil and extant pollen grains database

**Fig. 3:**
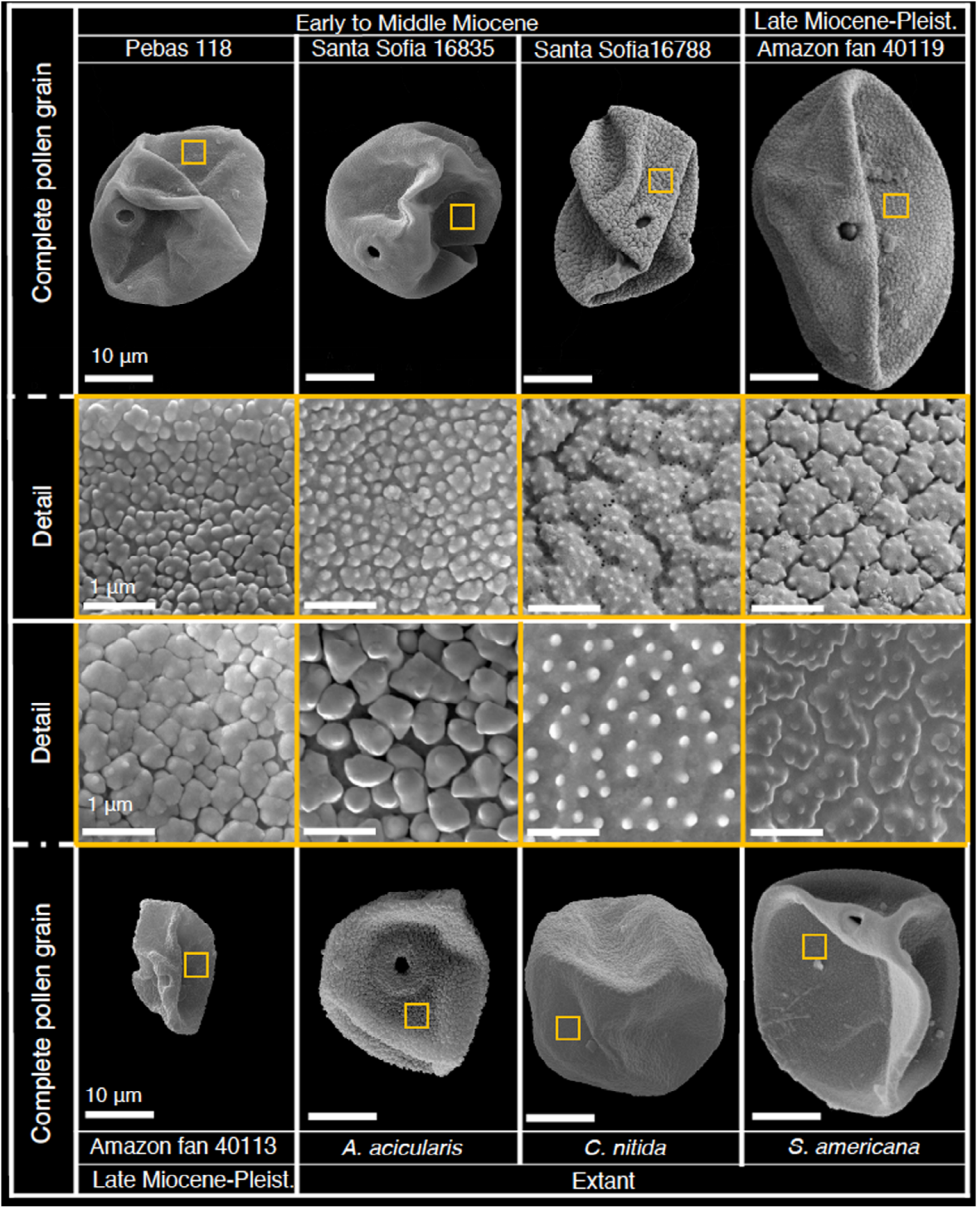
Representative specimens of Poaceae pollen grains from the Early Miocene to the Present. Group 1: fossil pollen of the Early to Middle Miocene from the western Amazon (Pebas and Santa Sofia sites); group 2: fossil pollen of the Late Miocene to the Pleistocene from the Amazon submarine fan; group 3: extant pollen (*Aciachne acicularis* Laegaard., *Cortaderia nitida* (Kunth) Pilg., and *Streptogyna americana* C.E.Hubb.) from South America. The upper and lower panels illustrate the complete pollen grains (Bars, 10 μm), the middle two panels show a surface segment. The precise location is marked by the orange box shown in the upper and lower panels (Bars, 1 μm). All images were taken under scanning electron microscopy.

We produced high‐resolution exine images of 1157 grass pollen grains of South America by using scanning electron microscopy. The database comprising 199 fossil grains of the western Amazon from the Early to Middle Miocene (the pollen count data from the samples are documented in Hoorn 1994); 155 fossil grains from the Amazon submarine fan and the Maracaibo Basin in Venezuela, ranging in age from the Late Miocene to the Pleistocene (up to *c*. 0.04 Ma) (the pollen count data from the samples are documented in Bermúdez *et al*. (2017) and Hoorn *et al*. (2017)); 803 extant pollen grains representing species from across grass phylogeny and ecosystems (the SEM images from the extant samples are documented in Wei *et al*. (2023); Fig. 3).

### Quantifying grass pollen morphology

We investigate the grass evolution in South America by conducting a comparative study of pollen morphology through space and time. Specifically, our study spans the Early Miocene to the Pleistocene fossil pollen and extant pollen from South America. Technically, we build on the work of Mander *et al*. (2013) and Wei *et al*. (2023), by utilizing 40‐dimensional vectors (size and density of pollen surface pattern and 38 features to quality the complexity of the surface) to quantify the surface ornamentation of individual pollen grains. This is the first application of the proposed technique to the analysis of fossil grass pollen. Our aim is to determine if the fossil and modern grass pollen have a comparable morphology and if their morphological space (from here onwards morphospace) changes over time. To achieve this, we carried out an extensive morphological analysis of fossil and extant pollen over time (Fig. 1a), using the SEM image library of fossil and extant grass pollen to provide a direct comparison (refer to Data Availability).

Quantitative analysis was conducted on 3.3 μm^2^ (1855 × 1855 pixel) windows, manually cropped from each 150 000× SEM image of grass pollen ornamentation. From each cropped image, 40‐dimensional vectors were extracted to evaluate the size, density, and complexity of pollen surface patterns. This method was adapted from the approach used for extant pollen developed by Mander *et al*. (2013) and Wei *et al*. (2023). Contrast‐limited adaptive bell‐shaped histogram equalization was applied for adjusting the contrast and then five sub‐images with 1000 × 1000 pixels were randomly chosen and cropped from each 1855 × 1855 image (Mander *et al*., 2013; Wei *et al*., 2023). All features were calculated as the average values extracted from these five sub‐images.

For the size feature, we employed Sobel edge detection and connected components to identify objects within the sub‐image, PCA was performed to measure the size feature (Wei *et al*., 2023). To calculate the density feature, we applied color quantization and selected the brightest component as the foreground, followed by morphological closing and then counted connected areas as the density of the sub‐image (Wei *et al*., 2023). All the detailed steps of construction of features size and density were available through previous research (fig. 2 of Wei *et al*. 2023). To assess the complexity of pollen surface patterns, we adapted the method which used the concept of subgraph centrality (SC) developed in Mander *et al*. (2013). In a previous study, SEM images were first coarsely classified into four groups based on size and density. Different groups used different methods to binarize the images: some used the top two brightest components as foreground, while others used the top three brightest components (Mander *et al*., 2013). However, the data used in this study has more continuous size and density features, which implies that the coarse classification is not suitable. Therefore we extracted SC features for both methods, top two brightest components (SC2) and top three brightest components (SC3) as foreground.

**Fig. 4:**
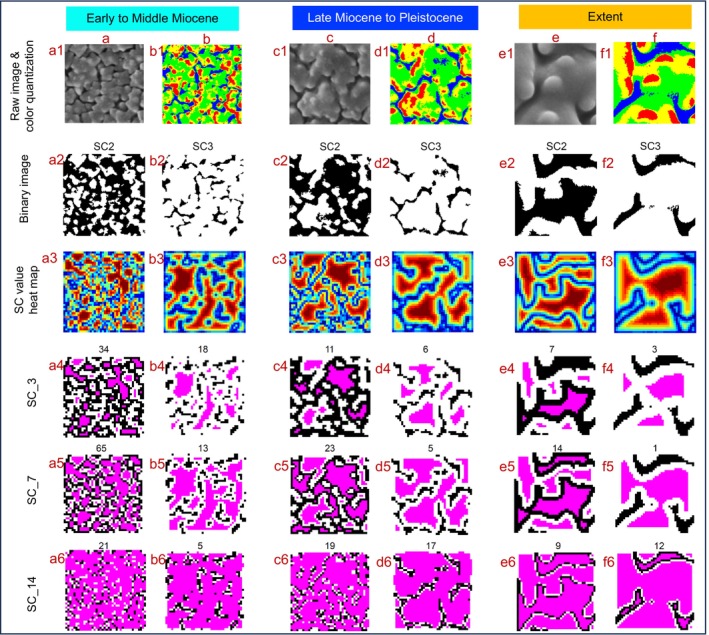
Thumbnails showing the example of image processing steps that were taken during the construction of SC2 features (a, c, e) and SC3 features (b, d, f). Example of grass pollen grains collected from the Early to Middle Miocene (a, b, from Los Chorros), the Late Miocene to the Pleistocene (c, d, from the Amazon submarine fan), and extant (e, f, *Bothriochloa saccharoides* (Sw.) Rydb.). Raw and quantized images (a1–f1): (a1, c1, e1) show randomly cropped raw sub‐images and (b1, d1, f1) show four colors quantization of raw images with brightness ordered from red to blue. Binary images for SC2 and SC3 calculation (a2–f2): (a2, c2, e2) show binary images with the foreground in white, derived using the 2 brightest colors (red and yellow) and (b2, d2, f2) show binary images derived using the 3 brightest colors (red, yellow, and green). These binary images are used for calculating SC2 and SC3 features, respectively. Subgraph centrality heatmap (a3–f3): the heatmap of subgraph centrality values (SC) with the largest value in red. SC features (a4–f6): the top 15% (SC_3, a4–f4), top 35% (SC_7, a5–f5), and top 70% (SC_14, a6–f6) pixels by ranking the SC values and are highlighted in pink, respectively. The number above each image represents the number of connected components of the pink region which are the corresponding SC2 and SC3 feature values.

For the detailed approach, we first resized the sub‐image with 1000 × 1000 pixels down to 200 × 200 pixels, and then cropped the center 120 × 120 pixels as the input image (Fig. 4a1,c1,e1). We then quantized each image into four colors (Fig. 4b1,d1,f1) and binarized it by using either top two brightest components as foreground (Fig. 4a1,c2,e2) or top three brightest components as foreground (Fig. 4b2,d2,f2). Then we further resized the image into 40 × 40 pixels to save computational cost and formed a network by connecting each pixel to its four neighbors. We assigned 0.01 to the edges which connect foreground and background. The rest edges are set to 1, then a mathematical function SC was calculated to rank the pixels (Fig. 4a3–f3). A sequence of 19 expanding subregions of the networks was formed, starting with the pixels ranked in the top 5% and adding the next 5% until the entire pixels was covered with number of connected component records as features (SC2_1 to SC2_19, or SC3_1 to SC3_19). Earlier features such as SC2_1 and SC2_2 describe the components with higher SC values which are more centered regions in the larger ‘chamber’. In the middle features such as SC2_7, many larger ‘chambers’ are connected by thin bridge and some median sized ‘chambers’ show up; later features like SC2_18 and SC2_19 exhibit almost entire region except lower SC values which are boundary pixels and smaller ‘chambers’. All the detailed steps of construction of features by using the subgraph centrality (SC) were shown in Fig. 4.

We automatically extracted morphological measurements from each fossil and extant grain directly from the SEM images for further analysis. The values of 40 quantitative features of each individual fossil and extant pollen grain are provided in Dataset S3 and S4, respectively.

### Morphometric analysis

#### Comparison of fossil and extant grass pollen grains

The quantified features of size and density values were logged before being used in data analyses, to make the data closer to a normal distribution. We utilized scatter plots of log size and log density for basic data visualization and exploration. Furthermore, PCA of 40 quantified features (log size, log density, 19‐dimensional features of SC2, and 19‐dimensional features of SC3) was applied to compare the morphospace of the fossil (groups 1–2) and extant pollen (group 3). Confidence ellipsoids were used to show the vector range of the PC1 and PC2 of fossil and extant pollen groups.

#### Exploration the grass pollen development through space and time

PCA was employed on all 40 quantified features to compare the morphospace of pollen from three spatial–temporal groups. The confidence ellipsoids show the vector range of the PC1 and PC2, and the Euclidean distance between the group centroids was calculated based on 40 features.

### Software

All image processing and feature extraction were carried out in matlab (R2017a). All data analysis and visualization were carried out using R v.4.1.1 (R Core Team, 2021).

## Results

### Comparison of fossil and extant Amazon grasses

**Fig. 5:**
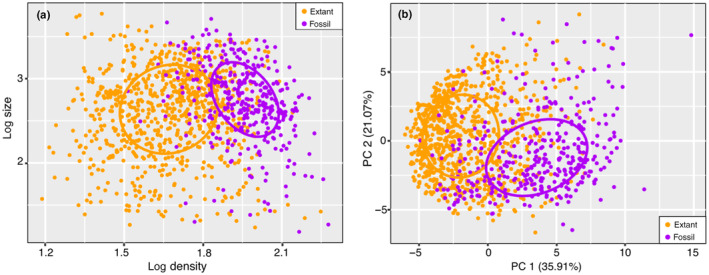
A comparison of the morphospace of grass pollen micro‐ornamentation between fossil samples (Miocene–Pleistocene) and extant samples from South America. (a) Scatter plot and confidence ellipses of fossil and extant grass based on log size and log density. (b) PCA plot and confidence ellipses of fossil and extant pollen using 40 quantitative features. The colored ellipses indicate the 50% confidence ellipsoids for fossil and extant pollen.

The quantitative measurements extracted from each individual fossil and extant pollen grain were used to construct morphospaces (Fig. 5). The preliminary exploration was conducted using a scatterplot of log size and log density of pollen surface elements, as well as confidence ellipsoids of fossil and extant groups (Fig. 5a). The scatter plot along with confidence ellipsoids show the distinct morphospace occupancy for fossil and extant grass pollen, while the position of confidence ellipsoids indicates that the values of log density in the fossil is higher than in the extant pollen. Our results suggest that the log density of grass pollen surface elements has gradually decreased through time.

The PCA of all 40 quantitative features (size, density, 19 SC2 features, and 19 SC3 features), along with confidence ellipsoids of PC1 and PC2, further confirms the distinct morphometric differences between fossil and extant pollen grains (Fig. 5b). PC1 and PC2 account for 35.91% and 21.07% of the total variation in all samples, respectively. The PC1 loadings shows the log density and the first 12 SC2 features (SC2_1 to SC2_12) contribute most, while first 13 SC3 features (SC3_1 to SC3_13) are contributing the most to PC2 (Fig. S1). The early and middle numbers in the SC series features driving the difference are dominated by the larger, thicker components, while higher numbers represent detailed or thin regions (see section on 'Quantifying grass pollen morphology'). The values of PC1 (dominated by earlier SC2 features) are generally lower/more negative for the extant grains, suggesting the pollen ornamentation (i.e. areoles, spinules) has gradually enlarged through time.

### Grass development through space and time

**Fig. 6:**
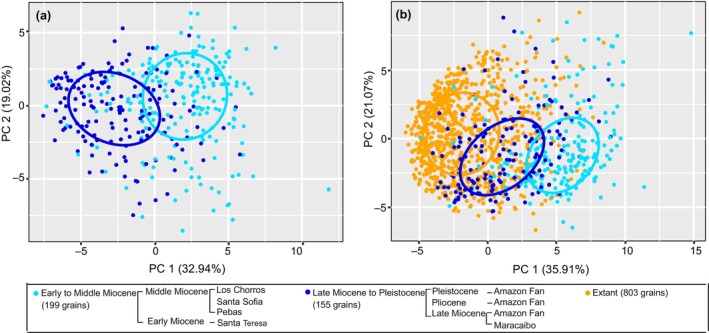
The morphospace and confidence ellipsoid comparison of grass pollen micro‐ornamentation through space and time. (a) PCA plot and confidence ellipsoids of the range of PC1 and PC2 showing the comparison of morphospace between the two groups of fossil samples. (b) PCA plot and confidence ellipsoids showing grass pollen morphospace between two groups of fossil pollen and extant pollen (note that this is the same PCA as in Fig. 4(b), but with the fossil points separated into two groups). The colored ellipse indicates the 50% confidence ellipsoids for the three pollen groups.

The PCA plots and confidence ellipsoids of PC1 and PC2 based on 40 quantitative features are utilized for both the groups of fossil pollen grains and the entire database (Fig. 6). For the PCA of just fossil groups (Fig. 6a), there is a distinction of morphospace occupancy between fossil pollen from the western Amazon of Early to Middle Miocene age (cyan points), and fossil pollen from the Amazon submarine fan and the Maracaibo Basin in Venezuela (dark blue points), dated as the Late Miocene to the Pleistocene. The first two axes of a PC account for 39.67% and 22.1%, respectively. The main contributing vectors to PC1 are SC2_9 and SC2_8, while SC2_5 and SC2_4 contribute to PC2.

When taking extant pollen into account (Fig. 6b), three spatiotemporal groups occur with unique and partially overlapping morphospace occupancy. Even though the pollen samples were derived from three different spatial–temporal groups, their extent – referring to the size of morphospace occupancy – did not obviously change over time. Regarding the location of morphospace expansion, there is a consistent morphometric (evolutionary) direction of change over time from higher to lower PC1 values, indicating a decrease in density of pollen surface micro‐ornamentation. This shift is noticeable from the Early to Middle Miocene interval (cyan), to the interval of the Late Miocene to the Pleistocene (dark blue), and then to the extant interval (orange).

The details of the PCA (percentage variance accounted for by the first two axes, and the main contributing vectors) are the same as those shown in Fig. 3(b). While calculating the Euclidean distance between the group centroids in ordination space between the three groups, the results showed that the distance between the Early to Middle Miocene group and the Late Miocene to the Pleistocene group is 4.57, and the distance between the Late Miocene to the Pleistocene group and the extant group is 3.3. This suggests that the Late Miocene to the Pleistocene group is closer to the extant group, when compared to the Early to Middle Miocene group.

When comparing the morphospace between fossil groups and extant pollen at the subfamily level (Fig. S2), the subfamilies Anomochlooideae and Pharoideae, which represent early diverging monophyletic groups, are closer in morphospace occupancy to the fossil groups.

## Discussion

### Potential drivers of change in Poaceae pollen wall morphology

Our study indicates that grass pollen assemblages from the Early to Middle Miocene interval, the Late Miocene to the Pleistocene interval, and present taxa each had unique but partially overlapping morphospace occupancy. The morphospace expansion of grass pollen shows a change in location rather than extent though time (Fig. 5b). When considering the rate at which grass pollen changed in terms of morphospace occupancy over time, we observe that the Euclidean distance between the centroids of confidence ellipsoids from the Early‐Middle Miocene group to the Late Miocene–Pleistocene group is 4.57, while the distance to the extant group is 3.3 (Fig. 5b). The relatively small difference in distances suggests a stable speed, or modest reduction, in evolutionary rate of change in pollen micro‐ornamentation that has been maintained over the observed time scale.

When revisiting the four scenarios that we proposed earlier, our morphospace data of the three intervals presents not distinct groups but rather unique and partially overlapping regions, which is most closely aligned with Scenario 2 (Fig. 2b). This scenario suggests that stable directional selection or drift (i.e. an evolutionary process) led to a gradual change in pollen morphology. Additionally, our study did not support the other three scenarios in terms of changes in the extent of morphospace (Fig. 2a), in the extent and location of morphospace occupancy (Fig. 2c), or no change in either extent or location of morphospace occupancy (Fig. 2d).

We suggest that there are three possible mechanisms that could explain change in grass pollen morphology through space and time.

#### Chemical and/or physical alteration of the pollen wall due to postdepositional diagenetic processes

Sporopollenin constitutes the outer wall of both pollen and spores (Gray & Boucot, 1971; Jaramillo *et al*., 2006; Mackenzie *et al*., 2015). A previous study indicated that the chemical composition of sporopollenin can be affected by early diagenetic processes (Jardine *et al*., 2021). This has drawn our attention to whether pollen micro‐ornamentation patterns can be influenced by chemical and/or physical alterations resulting from postdepositional processes.

**Fig. 7:**
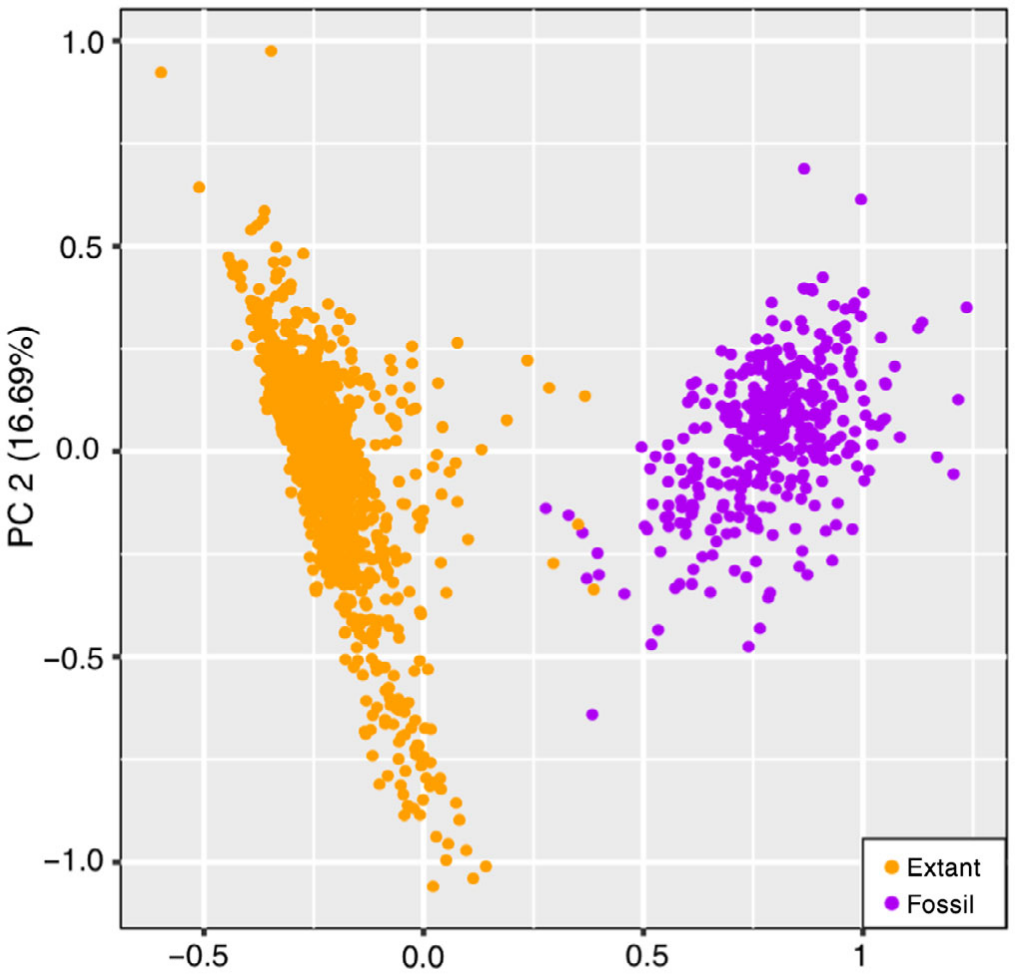
Re‐plotted PCA of FTIR spectra data from grass sporopollen throughout space and time (fig. 4a of Jardine *et al*., 2021).

In an earlier study, in which applied Fourier transform infrared (FTIR) microspectroscopy was applied to grass sporopollenin (Jardine *et al*., 2021), and in the present study, fossil pollen was both taken from the Amazon submarine fan and the western Amazon (the Early Miocene to the Pleistocene), and extant pollen from South America grasses. Jardine *et al*. (2021) showed that fossil and extant pollen in an ordinated chemospace (Fig. 7). If we envision that the pollen micro‐ornamentation patterns can be affected by chemical and/or physical alteration, the diagenetic effects should lead to a clear separation between extant and fossil, as seen in the chemistry (Fig. 5); however, this is not in agreement with our pollen morphometric data (Fig. 3a,b).

Moreover, the grain surfaces patterns in the fossil and extant pollen showed similar and clear patterns, such as areolae and/or spinules (Fig. 3). This all suggests that micro‐ornamentation, and thus physical appearance of the Poaceae pollen, is not compromised by postdepositional diagenetic processes. Our quantitative image analysis thus indicates that combining high‐resolution imaging of grass pollen on both fossil and extant grains is a robust way to assess changes in the grass pollen exine, which will enable us to assess, to some degree, the evolutionary function in grass pollen ornamentation across space and time.

#### Did abiotic change over space and time cause variation in pollen micro‐ornamentation?

**Fig. 8:**
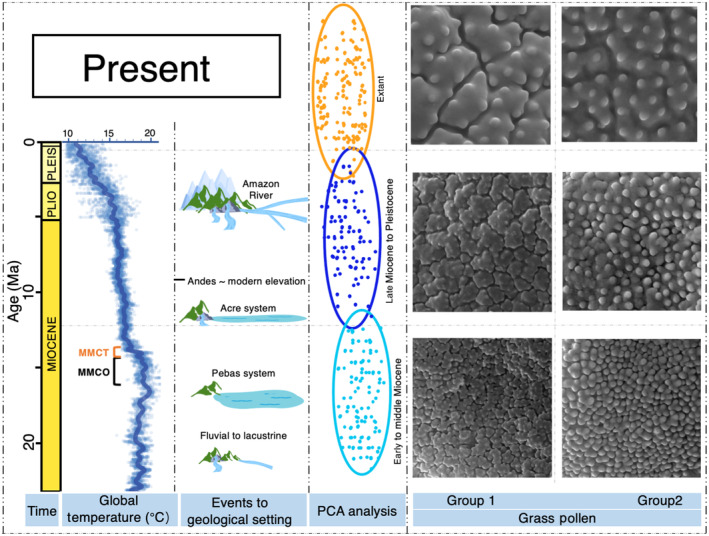
Grasses development scenarios through space and time in South America. From left to right showing: the age in million years; global surface temperature curve since Neogene (modified after Hoorn *et al*. (2023)), MMCO (Middle Miocene Climatic Optimum, *c*. 16.9–14.7 Ma), MMCT (Middle Miocene Climatic Transition (MMCT), *c*. 14.7–13.8 Ma); overview of events to three different geological settings in northern South America (Hoorn *et al*., 2010); schematic diagram illustrating the shift of pollen morphometrics from three geological setting groups over time, represented by confidence ellipses in Fig. 5(b); two sets of grass pollen surface segments with similar morphology were selected from the three geological settings: group 1 includes pollen surfaces within areolae studded with one to ten small pointed spinules; group 2 includes single spinules or small areolae studded by one to three spinules.

The development of grass biomes is thought to be linked to tectonism (Kohn & Fremd, 2008), climate (Edwards *et al*., 2010), and the combination of landscape dynamics and climate (Hoorn *et al*., 2023; Jaramillo, 2023). In this study, we divided our grass pollen samples into three spatiotemporal groups through the Neogene in South America, that are related to a series of geological events or settings (Fig. 8). Most noticeable in this sequence is the disappearance of the megawetland (i.e. ‘Pebas system’) in the western Amazon lowlands (*c*. 23–9 Ma) (Wesselingh & Salo, 2006; Hoorn *et al*., 2010). This wetland transitioned into a fluvial landscape, i.e the Amazon River, with extensive flood plains (*c*. 9–0.5 Ma) (Wesselingh & Salo, 2006; Hoorn *et al*., 2017). If we envision that the grass pollen morphology was affected by paleogeological events and/or paleoclimate in a certain period, we expect that some related restriction or expansion of morphospace occurred through time (Fig. 2a). However, our results show three grass pollen groupings with unique morphospaces occupancy, their extent has remained consistent through time (Fig. 4b). This also further confirms that the micro‐ornamentation morphospace of grass pollen is not driven by cross‐spatiotemporal abiotic factors.

Recent studies on extant grass pollen morphology have demonstrated that the quantitative features (size alone or density as well as their combination) of micro‐ornamentation patterns of grass pollen are not associated with climate, vegetation types, soil types, and photosynthetic pathways (Wei *et al*., 2023). In comparison to previous research, the extracted quantitative features of pollen micro‐ornamentation were here increased from two (size and density in Wei *et al*. (2023)) to 40 (size, density, 19 SC2 and 19 SC3). But, it should be noted that until now only a fraction of the total grass pollen flora has been tested for this relation. Our results show three grass pollen groupings with unique morphospaces occupancy, and their extent has remained consistent through time (Fig. 6b). This also further confirms that the micro‐ornamentation morphospace of grass pollen are not driven by cross‐spatiotemporal abiotic factors.

#### Evolution of pollen wall morphology

The PCA analysis indicated that the morphospace expansion of grass pollen shows a change in location rather than extent through time, suggesting pollen development towards a less dense ornamentation through time, with denser ornamentation types absent from our extant samples (Figs 5, 6, 8). We conclude that the changes in the exine of grass pollen since the Early Miocene would have led to a directional change in pollen morphospace occupation. We envision that the potential drivers caused the directional change in morphospace occupancy below.

##### Migration and other evolutionary processes

We propose the shift in pollen morphology resulted from the immigration of new taxa in or old taxa out through emigration. Additionally, the extent of morphospace occupancy, in terms of the range of morphological variation, remained consistent over time. Other evolutionary processes, specifically in evolutionary drift and/or directional selection, were/was expected to lead a directional development of pollen with less dense ornamentation over time.

##### Functional morphology

Changes in functional morphology of the Poaceae pollen wall could be a very plausible and powerful driver. The shift in morphospace occupancy observed in Poaceae pollen over time suggests an evolutionary advantage favoring lower density ornamentation. The evolutionary mode could potentially play a broader role, as a functional morphology altering the pollen ornamentation, contributing to the arrangement of taxa in morphological space and/or ecological significance in pollination (Ackerman, 2000; Traverse, 2007). For instance, the iterative or convergent evolution of pollen morphology might contribute to the differentiation of numerous subfamilies in evolutionary history (Traverse, 2007). Moreover, the majority of Poaceae species are wind‐pollinated, the evolutionary mode observed over time may benefit their development in wind‐pollination strategy (Linder, 1998; Mander *et al*., 2020). It would thus be of interest, to further investigate if there are evolutionary changes in the pollen wall over time that coincide with coeval changes in other plant organs (i.e. the devolvement of flowers or stigmas for pollen capture) within the Poaceae family (Niklas, 1985; Friedman & Barrett, 2011).

### Poaceae pollen wall evolution in the wider context of grass ecosystem evolution

At a global scale, diversification rates in the grass family were low during the Paleogene (*c*. 66–23 Ma), while increasing significantly during the Neogene (*c*. 23–5 Ma), which may be related to decreasing atmospheric CO_2_ (Palazzesi *et al*., 2022). We note that within our dataset of pollen morphological changes, a gradual, rather than punctuated, development occurs since Neogene for South America. These data overlap in time with the Neogene global expansion and diversification of grasses and grasslands that are highlighted by (Palazzesi *et al*., 2022). If this morphological signal is replicated across the globe (i.e. the pattern reflects evolutionary process alone) before the Neogene, this would suggest a decoupling of Poaceae pollen morphological evolution and global climatic change.

Notably, the diversification of the Poaceae pollen exine, and the trend toward a lower density distribution of the micro‐ornamentation, coincided in time with the exine revolution observed in Asteraceae (fig. 5b of Jardine *et al*. (2022)). Although recent studies suggest no apparent relation between abiotic factors and pollen morphology, the fossil record indicates that CO_2_ changes in the Neogene seemingly had a huge impact on pollen exine evolution (fig. 3a of Palazzesi *et al*. (2022)). It is plausible that Poaceae responded to the decrease in atmospheric CO_2_ by the evolution of pollen wall morphology at the micro‐ornamentation level, and this could explain micro‐ornamentation diversity patterns and changes in ornamentation density during the Neogene to Present.

We conducted a broad and deep‐time scale study of grass pollen to examine pollen development within the Poaceae family, and assess the underlying drivers of change since the Neogene in South America. Our study presents a novel approach to quantify fossil and extant pollen morphology by analyzing SEM images of pollen surface ornamentation using computational image analysis. Our research reveals that the morphospace occupancy of grass pollen shows a shift in location, but not extent, over time. The shift in morphospace can be characterized as a progression towards less dense ornamentation types. By exploring the underlying drivers behind shift in the pollen morphospace, we suggest that grass pollen evolution is linked to gradual directional change. We further propose that evolutionary processes (specifically in evolutionary drift and/or directional selection) and/or migration have played roles in the evolution of grass pollen morphology from the Early Miocene to the present in South America, and the functional morphology of pollen exines could be a plausible and powerful driver. Our findings suggest a gradual, rather than punctuated evolution of grass pollen since *c*. 23 Ma.

## Competing interests

None declared.

## Author contributions

CW, L Mander, PEJ, WDG and CH conceived and designed the research; CW collected herbarium specimens and CH collected fossil samples. CW, L Mao processed palynological samples for scanning electron microscope (SEM) experiments. CW performed SEM experiments. CW collected the SEM images and ML conducted the quantitative image analysis. CW carried out data visualization with contribution from PEJ, ML, L Mander, WDG and CH. CW wrote the first draft, all authors revised and approved the manuscript.

## Supporting information


**Dataset S1** Modern samples information.
**Dataset S2** Fossil samples information.
**Dataset S3** 40 quantitative features extracted from fossil pollen images.
**Dataset S4** 40 quantitative features extracted from modern pollen images.

**Fig. S1** Bar charts of component loadings for PC1 and PC2 from PCA analysis of grass pollen morphospace occupancy over time.
**Fig. S2** The morphospace and confidence ellipsoid comparison of grass pollen micro‐ornamentation on extant grass (subfamily level) and fossil grass (two spatial–temporal groups).
Please note: Wiley is not responsible for the content or functionality of any Supporting Information supplied by the authors. Any queries (other than missing material) should be directed to the *New Phytologist* Central Office.

## Data Availability

The original SEM images of fossil grass pollen image, MATLAB code for quantitative image analysis; the metadata and R code for data analysis for this study can be downloaded from: https://figshare.com/s/111a95932f929f06db9d. The original SEM images of modern grass pollen image can be downloaded from previous publication: doi: 10.6084/m9.figshare.23302022.v2.
