# Supplementary material for: A 23‐million‐year record of morphological evolution within Neotropical grass pollen
Source: New Phytol. 2024 Oct 27;246(1):365–76. doi: 10.1111/nph.20214 (PMC11883047; doi:10.1111/nph.20214)
Supplement: Supplementary file 2 — Fig. S1 Bar charts of component loadings for PC1 and PC2 from PCA analysis of grass pollen morphospace occupancy over time. Fig. S2 The morphospace and confidence ellipsoid comparison of grass pollen micro‐ornamentation on extant grass (subfamily level) and fossil grass (two spatial–temporal groups). Please note: Wiley is not responsible for the content or functionality of any Supporting Information supplied by the authors. Any queries (other than missing material) should be directed to the New Phytologist Central Office. [file NPH-246-365-s002.pdf]

***New Phytologist* Supporting Information**

Article title: A 23 million year record of morphological evolution within Neotropical grass pollen

Authors: Caixia Wei, Mao Li, Limi Mao, Luke Mander, Phillip E. Jardine, William D. Gosling, Carina Hoorn

Article acceptance date: 1 October 2024

The following Supporting Information is available for this article:

Fig. S1 Bar charts of component loadings for PC1 (a) and PC2 (b) from PCA analysis of grass pollen morphospace occupancy over time.

Fig. S2 The morphospace and confidence ellipsoid comparison of grass pollen micro-ornamentation on extant grass (subfamily level) and fossil grass (two spatial-temporal groups).

Fig. S1

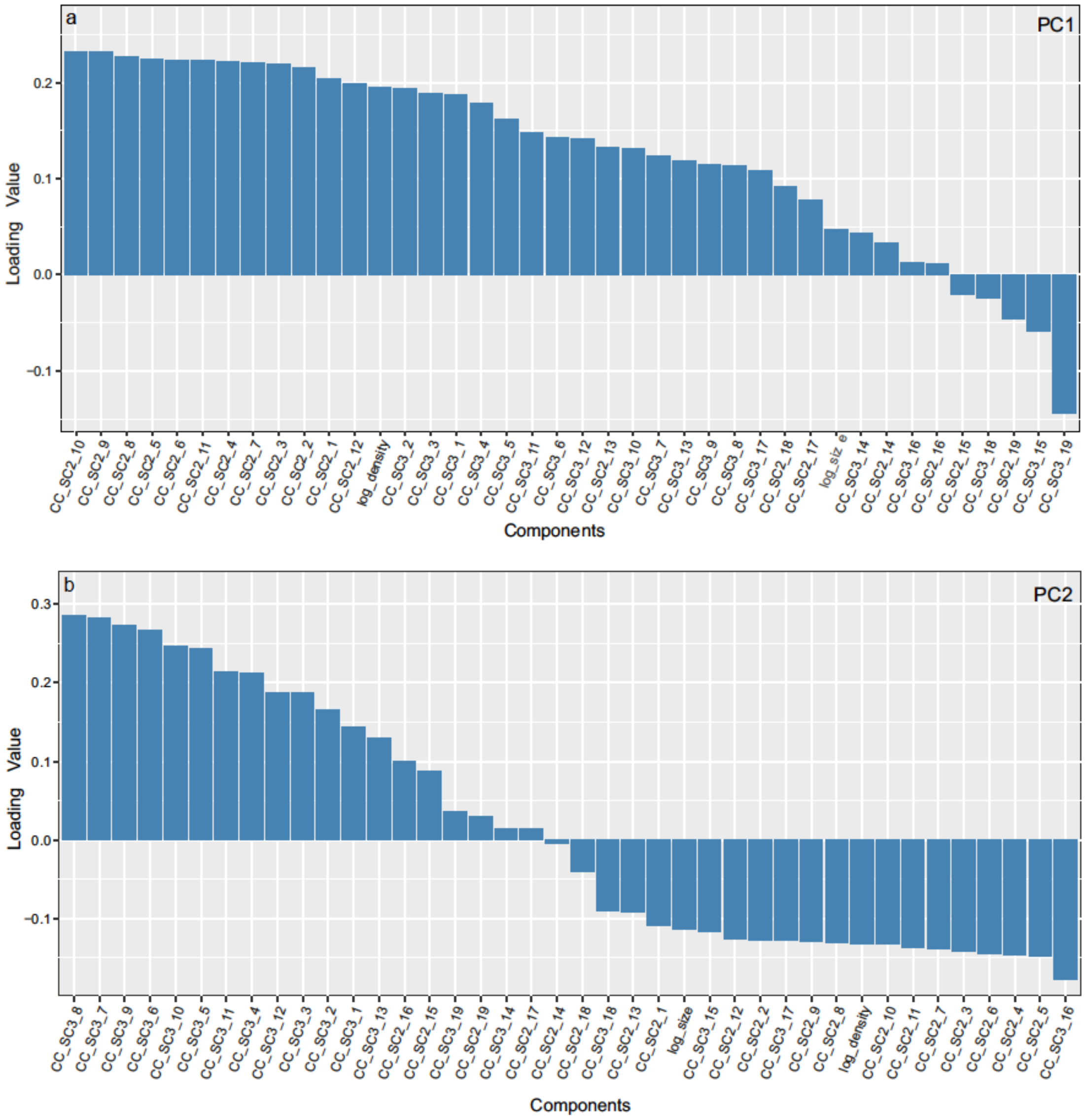

Fig. S1. Bar charts of component loadings for PC1 (a) and PC2 (b) from PCA analysis of grass pollen morphospace occupancy over time (Fig 3b; 4b of main manuscript).

Fig. S2

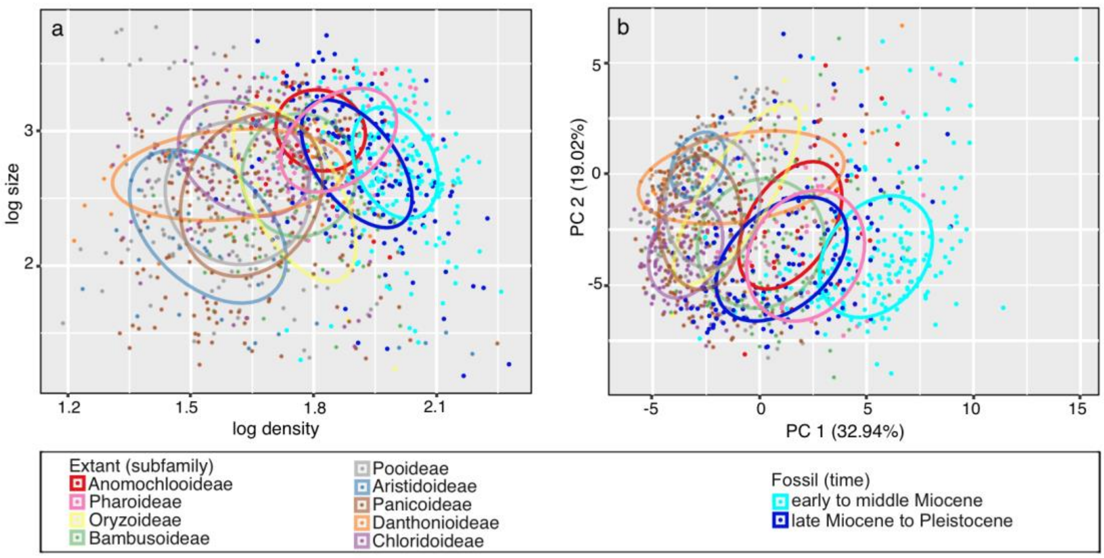

Fig. S2. The morphospace and confidence ellipsoid comparison of grass pollen micro-ornamentation on extant grass (subfamily level) and fossil grass (two spatial-temporal groups). a. Scatter plot and confidence ellipses of extant and fossil grass based on log size and log density. b. PCA plot and confidence ellipses of extant and fossil pollen using 40 quantitative features. The colored ellipses indicate the 50% confidence ellipsoids for fossil and extant pollen.
